# Opportunities for involving men and families in chronic disease management: a qualitative study from Chiapas, Mexico

**DOI:** 10.1186/s12889-015-2361-6

**Published:** 2015-10-05

**Authors:** Meredith P. Fort, Maricruz Castro, Liz Peña, Sergio Hernán López Hernández, Gabriel Arreola Camacho, Manuel Ramírez-Zea, Homero Martínez

**Affiliations:** INCAP Research Center for the Prevention of Chronic Diseases (CIIPEC), Calzada Roosevelt 6-25, Zona 11, Guatemala City, Guatemala; Department of Family Medicine, University of Colorado – Denver, Denver, CO USA; School of Nutrition, University of Sciences and Arts of Chiapas, Tuxtla Gutiérrez, Chiapas Mexico; RAND Corporation, 1776 Main Street, Santa Monica, CA USA; Hospital Infantil de México, “Dr. Federico Gómez”, Mexico City, Mexico

**Keywords:** Family relationships, Gender, Primary care, Type 2 diabetes, Hypertension, Self-management, Patient support, Prevention

## Abstract

**Background:**

A healthy lifestyle intervention was implemented in primary care health centers in urban parts of Tuxtla Gutiérrez, Chiapas, Mexico with an aim of reducing cardiovascular disease risk for patients with type 2 diabetes and/or hypertension. During implementation, research questions emerged. Considerably fewer men participated in the intervention than women, and an opportunity was identified to increase the reach of activities aimed at improving disease self-management through strategies involving family members. A qualitative study was conducted to identify strategies to involve men and engage family members in disease management and risk reduction.

**Methods:**

Nine men with hypertension and/or type 2 diabetes with limited to no participation in disease self-management and health promotion activities, six families in which at least one family member had a diagnosis of one or both conditions, and nine health care providers from four different government health centers were recruited for the study. Participants took part in semi-structured interviews. During interviews with families, genograms and eco-maps were used to diagram family composition and structure, and capture the nature of patients’ relationships to the extended family and community resources. Transcripts were coded and a general inductive analytic approach was used to identify themes related to men’s limited participation in health promotion activities, family support and barriers to disease management, and health care providers’ recommendations.

**Results:**

Participants reported barriers to men’s participation in chronic disease management and healthy lifestyle education activities that can be grouped into two categories: internal and external factors. Internal factors are those for which they are able to make the decision on their own and external factors are those that are not related solely to their decision to take part or not. Four primary aspects were identified related to families’ relationships with disease: different roles within the family, types of support provided to patients, the opportunity to prevent disease among family members without a diagnosis, and - in some cases - lack of family support or stress-induced by other family members. There was an overlap in recommended strategies for engaging men and family members in chronic disease management activities.

**Conclusions:**

There is an opportunity to increase the reach of interventions aimed at improving disease self-management by engaging men and family members. The proposed strategies presented by patients, family members, and providers have implications for health education and service provision at primary care health centers and for future research.

## Background

In recent years, Mexico has undergone a rapid epidemiological and nutritional transition leading to an increase in non-communicable diseases and obesity [1]. Chronic conditions place a major burden on the population and the health care system, as 31.5 % of Mexican adults have hypertension and 9.2 % have type 2 diabetes [2].

In response to the growing problem of non-communicable diseases worldwide, the United States National Heart, Lung, and Blood Institute (NHLBI) and the United Health Group funded a network of research centers globally to build research capacity and to conduct population-based and clinical studies focused on the prevention and management of cardiovascular and pulmonary diseases [3].

One of the studies funded through this initiative was a healthy lifestyle intervention for patients with type-2 diabetes and/or hypertension, implemented in primary care health centers in Chiapas, Mexico and San José, Costa Rica, that had an aim of increasing heart healthy behavior and patients’ capacity to manage their conditions. The intervention study in the Chiapas site showed an increase in stages-of-change activation among patients [4]. The study in Mexico and Costa Rica drew on a previous experience in an urban health center in Guatemala, that resulted in dramatic reduction in systolic and diastolic blood pressure comparing baseline and 6 months of having been exposed to the intervention [5].

While both studies demonstrated the potential that group education sessions have to encourage patients to manage their conditions, the studies also point to the importance of identifying the barriers that certain sub-groups face in participating in healthy lifestyle interventions as well as the opportunities that exist to increase interventions’ reach. During the intervention study that was rolled out from the end of 2011 to the end of 2012, there was very limited participation from men; in Chiapas, 92 % of the 95 patients enrolled in the intervention group were female. Low involvement by men is not unique to Chiapas. In San José, Costa Rica, while not as marked, 40 % of participants in the intervention group were male. In a previous study in Guatemala, only 29 % of study participants were men. While gender differences in the uptake of health promotion interventions is a documented phenomenon, gender-tailored approaches are often not implemented [6].

Patient engagement in chronic disease management is increasingly considered to be important to improving patient outcomes; providers are recognized as crucial in fostering patients’ engagement [7] and each individual patient's process for assigning meaning to their condition also influences their engagement [8]. Specifically, studying patient sub-groups that are less engaged may provide insight into how to tailor approaches to better reach them.

The research team also identified, during focus group discussions in the formative research phase, that there was an opportunity for a family-oriented approach, rather than an intervention focusing on individuals with one or more condition. In an analysis of the focus group discussion transcripts, the role of family was one of the key factors mentioned, both as a facilitator for and a barrier to disease self-management [9]. During the healthy lifestyle intervention, on occasion, family members showed up to accompany individual participants selected for the intervention, highlighting the opportunity to define alternative strategies to also reach family members who were dedicating time to take part in the health education sessions, although they were not being tracked as participants.

There is growing evidence of the potential that strategies involving family members have to support a family member affected by a chronic condition [10–12]. Specifically, interventions focused on engaging family members may improve self-management support for patients who have poorly controlled type 2 diabetes [13]. However, family interactions may be both a facilitator for and a barrier to disease management [14]. In addition to the opportunity to support families with type 2 diabetes to reach their self-management goals, clinical practice may also stand to gain by reaching others at high risk for type 2 diabetes within the families of patients that already have a diagnosis. Having a biologically related relative with type 2 diabetes increases diabetes risk, and increased risk for spouses has also been documented [15]; given the shared risk between spouses, there is an opportunity to improve diabetes detection and motivate couples to increase collaborative efforts to optimize eating and physical activity habits. Family members who provide support for somebody affected by a chronic condition may be an important group to reach as offering support to others may influence their ability to take care of themselves.

While recognized to be an innovative topic with the potential to contribute to improved health outcomes, there is uncertainty about how best to involve family members in interventions. Often family-focused research and interventions focus on dyads - studying couples or parent-and-child pairs. However, family relationships as they relate to chronic disease are complex and multi-faceted. As there is no standard family composition, it is also important to understand the family as an entire unit; a review of the literature on family interventions among adults with diabetes found that because the family is often not included in interventions in a comprehensive way, the measurable effect of the “family” contribution is not easy to determine and is “lost in translation” [16]. Tools such as genograms and eco-maps have been used largely in clinical contexts but may also be useful in research, and may complement more commonly used interview methods [17].

In light of the new questions that arose during the implementation of the healthy lifestyle intervention, we conducted a final qualitative phase post-intervention to: a) understand the barriers that men with one or more chronic condition face in participating in health promotion activities, b) characterize family dynamics related to chronic disease management when somebody in the family has a chronic condition, and c) outline strategies that were proposed by patients, families, and providers to increase participation by men and to involve family members in activities aimed at reducing cardiovascular disease risk.

This article presents the findings from this study as a contribution to inform primary health care service providers so that they may develop health promotion strategies to reduce cardiovascular disease, with an aim of increasing participation by men and family members.

## Methods

From February 2013 to February 2014, researchers from the Institute of Nutrition of Central America and Panamá and the School of Nutrition at the University of Sciences and Arts of Chiapas in Tuxtla Gutiérrez, Chiapas, Mexico implemented a qualitative study to better understand ways to increase uptake and expand the reach of a NHLBI-funded cardiovascular disease risk reduction education model that had been previously implemented in urban primary care health centers in both Chiapas and Costa Rica and concluded in December of 2012.

The study team contacted Secretary of Health center staff in Tuxtla Gutiérrez to identify health care workers, male patients, and families to invite to take part in the study. Nine men with a diagnosis of hypertension and/or type 2 diabetes who had limited or no participation in health education programs were identified and recruited from primary care health centers of the Secretary of Health for in-depth, semi-structured interviews. One additional eligible man was invited to take part in the study but due to difficulty scheduling an interview did not participate. Six families in which at least one family member had a diagnosis of hypertension and/or type 2 diabetes who received care at a Secretary of Health clinic were identified and recruited to participate in the study. One additional family was invited to participate but was facing an unrelated health situation at the time of the study and chose not to participate. Nine health care providers from four different health centers of the Secretary of Health in Chiapas, Mexico were also interviewed for the study. We sought to include a range of provider types that interact with patients with type 2 diabetes and/or hypertension at government health centers. The health care providers included one medical health center coordinator, one physician, two nurses, three nutritionists, one social worker, and one health promoter. One additional nurse was contacted and invited to participate but due to scheduling difficulty did not take part in an interview. Health care workers were invited to participate to capture their perspective on the patient population they serve and to have a provider perspective on strategies that may be appropriate for engaging men and families. For each of the three sub-populations, the number included in the study was within the range of expected study participants outlined in the protocol, recognizing that the aim of the study was to explore themes and identify potential strategies to engage men and family members to be continually explored in the future.

### Data collection

All three sub-populations were interviewed in Spanish by one or both of the study team’s co-investigators, both of whom are members of the faculty of the School of Nutrition of the University of Sciences and Arts of Chiapas. Prior to initiating the study, the co-investigators tried out questions from the interview guide with individuals with similar characteristics to those included in the study in order to make sure that they were understandable. The specific content covered in the interviews were: disease self-management activities, barriers to participating in health promotion activities at the health center, changes within the household related to the chronic condition, purchase and preparation of food, types of accompaniment and support, and strategies for including family members and reaching men.

Individual interviews with men were conducted at the university, a health center, or in their home. The six family interviews were conducted in the families’ homes; in addition to the index family member (identified by the health center as having a diagnosis of hypertension and/or diabetes), at least one other adult family member residing in the same household was present and took part in the group interview. Interviews with health care providers were conducted individually, at the health center where each one worked. Each interview was audio-recorded and transcribed into a separate Word document in Spanish.

During the family interviews, in addition to a set of questions, two additional techniques were used: eco-maps and genograms. An eco-map is a tool for graphically portraying personal and family social relationships and a genogram is used to capture the composition and structure of a family. The eco-map was used to document the major systems that are a part of the family’s life and the nature of the relationship that the family has to the different systems [18]. The genogram was used to capture the complete list of family members, their disease status and history, and the nature of the family members’ relationship to one another that can present barriers or facilitation of disease management; genograms were created using a standardized set of symbols [19]. While these tools are used primarily for clinical purposes, a number of researchers advocate for the use of eco-maps and genograms in public health research [17, 20]. One genogram and one eco-map were drawn on paper during each family interview by a study co-investigator.

The study protocol was approved by the Institutional Review Boards of the RAND Corporation (Study #2010-0700), the Institute of Nutrition of Central America and Panama, and the Health Institute of the State of Chiapas. Written informed consent was obtained from all study participants; in interviews with adult family members, each participating family member signed their own consent form. Transcripts of interviews and ATLAS.ti-coded files are housed at the School of Nutrition of the University of Sciences and Arts in Chiapas, Mexico. Pseudonyms are used in this article and in study reports in order to ensure confidentiality.

### Data analysis

We used a general inductive analysis approach [21] to identify themes related to men’s limited participation in health promotion activities, how the family is engaged in disease management when somebody in the household has one or both conditions, and recommendations for incorporating men and family members into care and prevention of cardiovascular disease. We used the following stages in our analysis: familiarization with the data, generation of initial codes, searching for themes among codes, reviewing themes, defining and naming themes.

During the analysis process, all transcripts from interviews with men, families, and health care providers were entered in to ATLAS.ti version 7 (2013) qualitative data management software (Scientific Software Development, Berlin, Germany) and three Spanish-speaking analysts used open coding and focused coding to produce an initial list of codes that we analyzed collectively and converted into an agreed-upon set of codes. The coded transcripts were then reviewed by two other members of the research team for consistency in coding and the team as a whole resolved differences through group discussion.

A theme was defined as a “common thread that runs through the data” [22]. The research team identified quotes that best illustrated common themes and included them in the results of this study. Quotes were translated into English by bilingual authors and were edited only for ease of reading.

The genograms and eco-maps that were drawn on paper for the six families were laid out side-by-side and analyzed for interactions between family members, gender and support roles, and to explore resources that families draw on for disease management and cardiovascular risk reduction. The analysis of genograms and eco-maps was done in parallel to the coding and analysis process of interview transcripts.

We have followed guidelines for reporting qualitative research findings [23].

## Results

A number of different barriers were reported as explanations for why men have limited participation in chronic disease management and cardiovascular health promotion education activities. These barriers have been grouped into internal and external barriers and are presented in Table 1. Internal barriers are those which individuals have control over, and external barriers are those that do not depend solely on an individual’s decision, but also depend on factors for which they are not able to make a decision on their own.

**Table 1:** Barriers that men face in participating in healthy lifestyle group education sessions

Barriers	Topics	Specific explanations
Internal barriers	Time	- Lack of time
	Gender roles	- Belief that men have to be strong
		- Perception that activities are geared toward women
	Age	- Perception of not fitting in; others in the group are older
		- Too old to learn
	Perception of chronic condition	- Concern that if they found one chronic condition they might find another
		- Not inspiring to be with others who have your condition
		- Sufficient to go to a clinic visit with the doctor
		- Fatalistic view of disease
External barriers	Work	- Conflicting work schedules
		- Type of work that does not allow for committing to regular attendance
	Health center staffing, programs, and communication with staff	- Health center staffing is limited on weekends when men might be more likely to attend
		- Mostly female providers that may inhibit men from participating
		- Some health centers do not offer educational sessions
		- Under-resourced health centers turn people off from participating
		- Communication gap with provider about who may attend

### Internal barriers to men’s participation

#### Time

Time is listed as an internal barrier, which captures the extent to which an individual prioritizes participation, but may be an external barrier if somebody has limited control over their time. For example, a 54-year old man with type 2 diabetes and hypertension explains that he has not taken part in group education sessions because of available time, given his work schedule as a taxi driver: *“Because of my time, because I work in the afternoons and in the morning I focus on resting and other things.”* He then goes on to explain that on weekends he works long full-day shifts, preventing him from taking part in the group sessions offered then.

#### Gender roles

Gender was a barrier that was mentioned in terms of the perceived difference in men’s and women’s roles and how they respond to their condition. A 58-year old man with hypertension and diabetes highlighted pride and men not accepting their illness as factors for not participating: “… *pride, stubbornness, because there is not a man who accepts his illness, and even if he is falling over he says ‘I am fine’ and with his friends the same thing, pride*”.

In addition to men themselves presenting explanations, health care providers referred to men and women having different roles in society and that in general men take part in health center groups only when their condition is severe or if somebody in the family tells them that they have to. A nutritionist explained what she has observed: “*Men are the most difficult ones to involve in a treatment group, if they go for a clinic visit it is because they are forced to or because of a strong pain or because of something urgent, but all of the health units have the same problem that men are not involved, if they show up it is because of their wife or their son or daughter, but not of their own volition.*”

#### Age

The age of participants was mentioned as a potential barrier both in terms of feeling that one did not fit in because the other participants were older and also in terms of the perception that patients are too old to learn. A 47-year old man with diabetes felt that he was young as compared to others who were at the group sessions: “*Because, for example in my case, the majority I felt were older than me …*” A 54-year old man with hypertension and diabetes referred to comments he had heard from patients in reference to their age: *“I also think of people’s idiosyncrasies when they say: ‘I am too old to be learning.’”*

#### Perception of one’s chronic condition

In the interviews with men, how men perceived their chronic condition at times represented a barrier to participation. A 69-year old man with diabetes and hypertension explained that he did not consider it inspiring to be with others with his chronic conditions: “*Sharing with people who have your same problem, does not really inspire you, and I prefer to consider myself to be on the healthy side and I try to work on it in my own way, in private and alone.*” In some cases, patients described their condition in fatalistic terms, stating that all people have to die from something.

Another explanation provided by a health promoter was that it was perceived to be sufficient to go to check-ups with a doctor: *“The men who do not attend (health promotion sessions) and have diabetes it is because they have the idea, well I am already going to check-ups with my doctor, and I have my condition under control, it isn’t necessary for me to show, and you think “ah, ha”.*

### External barriers to men’s participation

#### Work

Men reported difficulties participating in health education sessions both because of work schedules that conflicted with the timing of the sessions, and also because of the type of work that did not allow them to plan when they would or would not be working, as is the case for contract or seasonal jobs.

Health care providers also noted work as a factor limiting men’s participation. A nutritionist drew on her experience working with male patients to explain how work can prevent them from taking part in group sessions organized by the health center: “*And he told me that he was a carpenter and well he does not keep regular hours but has to stick with working certain days, so I think that has been the primary obstacle that patients – male patients – face in taking part in the mutual support group.*

#### Health center staffing, programs, and communication with health center staff

Another type of barrier that men and health care providers reported was related to health center staffing, program offerings, and communication. Providers explained that on weekends staffing is more limited, which is a time when men might be more likely to attend. Men mentioned that having more female than male health care workers in the area of chronic disease health promotion was also a factor inhibiting their participation. A range of offerings were reported at the different centers with some having active programs and others that did not. And the communication gap between health center staff and patients about eligibility to participate was the explanation that one 64-year old man with hypertension gave for why he did not participate. He explained: “*I went once, since they had a meeting but she told me – yes, she was the one who told me - that the meetings were just for diabetics.*”

### Characterization of family engagement when somebody has one or more chronic condition

Below is a characterization of four aspects of family engagement when somebody in the family has one or more chronic condition.

#### Roles within the family

A number of roles within the family were identified that are relevant for understanding how individual family members and the family as a whole manage the chronic condition. Roles are largely identified along gender lines and in some cases by generation within the family. The key identified roles were: communicator (both externally and internally, that was primarily considered to be a woman’s role), wage earner (that was largely a man’s role), caretaker (that was largely a woman’s role), and food preparer (that was largely a woman’s role).

Below are examples of women taking on the role of preparing food for others in her family. While interview participants mostly spoke of men assuming the wage earner role, the quote below also shows that women – especially in the younger generation – are part of the workforce assuming this role as well.

“*Yes because since I work I leave their breakfast and meals ready and they just heat it up.”* (daughter of a 60-year old man with diabetes).

This second example highlights that in some cases family members not living in the same household will assume the role of food preparation. Interviewer: *“So, you are in charge of the meals for the whole family.”*

Wife (41 years old): *“Yes.”*

Interviewer: *“And when you are not there, who takes your place?”*

Husband (45 years old, with hypertension and obese): *“Her sister.”*

Wife: *“My sister, my sister comes to prepare something, but mostly I leave it all ready.”*

#### Support for the diagnosed family member

Five categories of types of support for a family member with the diagnosis of hypertension and/or type 2 diabetes were mentioned during the interviews: food preparation and purchase, transport or accompaniment to the health center for a clinic visit or an educational session, obtaining medications, economic support, and emotional support.

One of the primary sources of support that was mentioned was food preparation and purchase. A 60-year old man with diabetes describes the support that he receives from his wife and ends by saying that she also began to eat vegetables:

“… *it depends on me and also my wife who prepares things for me, she brings me green beans, she brings me squash, she brings me carrots, she brings me umm yucca or bags of vegetables, she just cooks it and that is what I eat, and even she is starting to too she says*.”

**Fig. 1:**
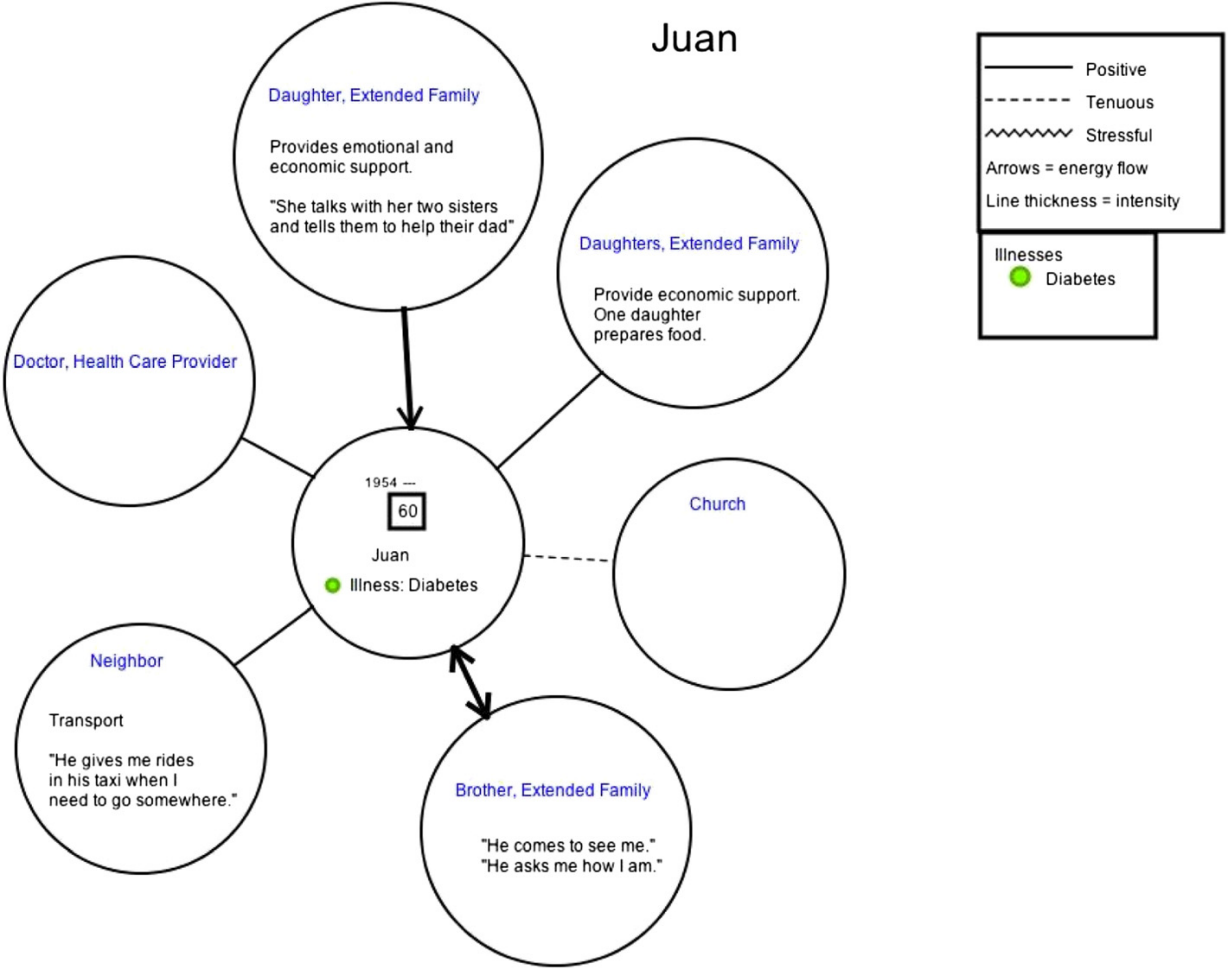
An eco-map of a 60-year old male patient with type 2 diabetes

The use of eco-maps was helpful for identifying individual people who provide support and providing specific examples of the types of support that index family members receive, both from the broader community and also from extended family members. For example, in Fig. 1, an eco-map, for a 60-year old male index member who we will call Juan who has type 2 diabetes. Juan has identified his primary source of support to come from one of his daughters and from his brother. The specific types of support he mentioned included: economic, emotional, food preparation, transport, and generally being available.

#### Opportunity presented when somebody in the family has a chronic condition

A number of family members and patients with one or more of the chronic conditions of interest mentioned that having the diagnosis provided an opportunity for the family. The opportunity was identified through improved diet for all family members, allowing the diagnosed family member to educate other family members about either or both conditions, and the recognition of emotional needs and improved communication.

“*I have always had family support and it is really important because it is also a way for them to learn, even if they are not suffering from a condition they can learn how to avoid having it, yes that is right* (man with diabetes and hypertension, 54 years old).

**Fig. 2:**
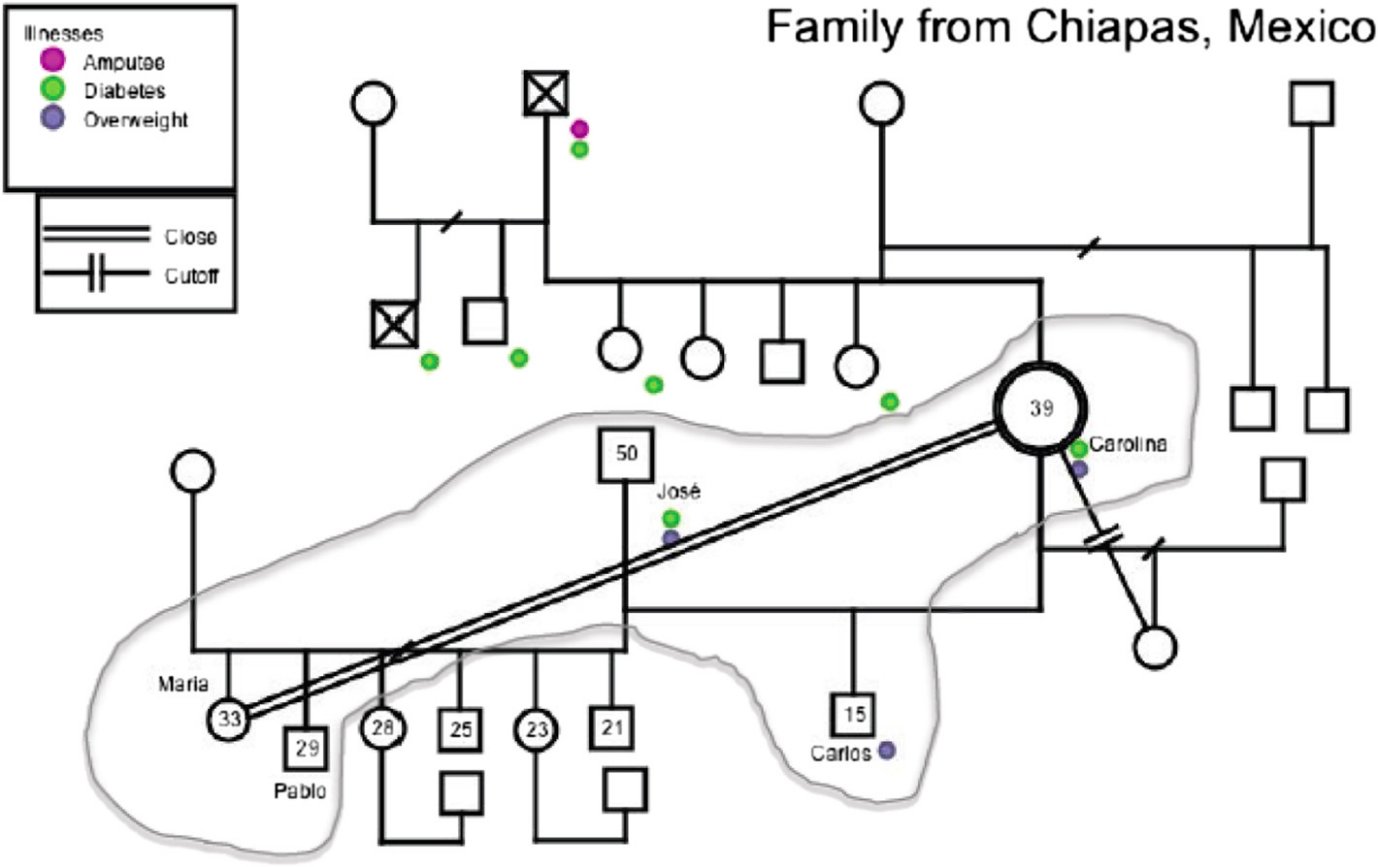
Genogram for a family in which the index family member is a 39-year old woman with type 2 diabetes

Figure 2, a genogram for one of the families that was interviewed in the study, represents this potential opportunity. The gray line encircles family members residing within the same household. Both the husband and wife, represented in the genogram, have diabetes and are also overweight. In this example, 39-year old Carolina (pseudonym) is selected to be the index family member with a thicker-outlined circle, but her 50-year old husband José (pseudonym) could have been the selected index family member as he also has type 2 diabetes. Given that there are multiple family members living in the same household with diabetes, as well as others in the extended family, individual family members may support others with a condition and be patients at the same time, or may have alternating roles at different times during the course of their disease.

During the study, the research team recognized that genograms may provide information to clinicians and researchers, and also may be a tool for family members to visualize how the disease has affected the family and what the potential risk of disease is for an undiagnosed family member. For example, seeing the 15-year old boy Carlos (pseudonym) with two diabetic parents and recognizing that he is currently overweight presents an opportunity for health center staff who interact with his mother, and also for the family itself, to define strategies to promote healthy behavior within the household.

#### Lack of family support

In some cases, particularly in interviews with providers, lack of family support for an individual diagnosed with hypertension and/or type 2 diabetes was considered to be a problem for an individual's self-management. Specifically, concerns that were mentioned were individuals who live or eat meals alone, individuals who are dependent on other family members but may not get support in disease management, stress induced by family members, and a general sense of being alone in their experience with the disease.

For example, the daughter of a 70-year old woman with hypertension began by explaining techniques her mother has used to keep her blood pressure under control, and how the home environment may affect her in a negative way: “*Walking and talking because that way she keeps busy and her blood pressure stays calm, on the other hand if she is here inside, we have kids and the kids are running around, one cries then the other one, and because she is older and she has high blood pressure well it is affected…*”

A nurse identified older adults who live alone as a group that lacks family support: “…*I have realized that because they live alone, I mean there is an abandonment, self-care is important and not so much from a family member, as much as we would like to have a family member intervene to help the patient take their medications, limit the sugars they eat, and eat more fruits and vegetables*…”

#### Strategies for involving men and families in health promotion activities

The primary strategies proposed by men, families and health care providers to increase participation by men and families in health education activities aimed at reducing cardiovascular disease risk are presented in Table 2.

**Table 2:** Proposed strategies to involve men and family members in cardiovascular disease risk reduction health promotion activities

Involving men	Involving family members
Offer sessions specifically for groups of men rather than making them co-ed	Offer sessions specifically for family members
Offer sessions on weekends when men are more likely to not be working	Offer programs on weekends when families are more likely to be able to attend as a group
Offer activities specifically for couples as men often are encouraged to attend by their partners	Invite families to participate in health education sessions and/or to accompany a family member with a chronic condition to their clinic visit
Allow for drop-in opportunities rather than projecting participation in all sessions to be obligatory	Incorporate home visits into routine practice
Have activities at group education sessions that resonate more with men	Adopt a strategy that motivates family members to have contact with the Secretary of Health, (e.g. similar to the government’s cash transfer program)
Have more male health care providers involved in the sessions to make the environment more inviting to men	

The strategies to increase involvement from men included: offering sessions just for men with activities targeted at men, offering sessions for couples, scheduling sessions on weekends when men are less likely to be working, allowing for drop-in opportunities rather than making participation in all sessions required, and involving more male health care providers. A man with diabetes referred to the possibility of offering sessions for men: “…*it would be better for me, because when you are there with women they always begin to talk about other things and instead with men you all talk together… you feel more comfortable.*”

The strategies to increase family engagement included: offering sessions for family members, scheduling weekend sessions, having health care providers invite family members, incorporating home visits into health center programming, and motivating family members to participate through strategies such as a government cash transfer program.

A nutritionist proposed making changes to offer more weekend sessions as a way to increase involvement from men and families, and recognized that the timing and the approach would need to be different: *“… create a schedule that is more possible for families and men to participate, for example it could be Saturdays in the afternoon or Sunday mornings and include activities for the whole family, for example the majority of families have 5 or 6 people so… a strategy would be to invite three families to not have so many patients here at the center…”*

Family visits were recommended by men, families, and health care providers. A nutritionist working at one of the primary care health centers described the advantage of this strategy as it allows her to be in contact with the population seeking care through a safety net provider, in contrast with other institutions.

“… *Here in the Secretary of Health, I feel that one works a little closer to the patient, because we also have activities in the field where we go out and visit patients, because it is not the same to receive them here as it is to really see how they are living, and under what conditions.*”

An experience during this study highlights the potential that this strategy may offer to understand patients’ reality beyond what can be captured during a clinical encounter or a group education session. During a family interview with the family of a 70-year old woman with hypertension who had participated in the health education intervention during 2012 and who regularly receives care at her designated Secretary of Health clinic, the research team realized that the patient does not take her hypertension medication.

Her daughter explained: “*What happens is that she does not tell the doctor because he scolds her… every month they give her a pill, but she does not take her blood pressure pill because if she does then what affects her is her heart.*”

The patient had not found an adequate time to talk about her decision to not take her medication during her consultations with her provider and also did not mention it in any of the group education sessions as one of the topics presented in the sessions was the importance of treatment adherence. While the interview was not a programmed family visit, the opportunity to have the family present provided a moment in which the other family members talked openly about how their mother did not take her medication.

## Discussion

The primary findings from this study are that men face internal and external barriers that limit their participation in cardiovascular disease risk reduction education activities and that there is an opportunity to involve family members both in a support role and also as a way to prevent disease.

There is an overlap in strategies aimed at involving men and families in cardiovascular health promotion activities at the primary care delivery level. As men in Chiapas are more likely to be employed than women [24], strategies related to the timing of program offerings are particularly important, recognizing that this societal factor may restrict men more than women in their participation in healthy lifestyle promotion sessions offered at health centers. The proposed strategies made by men, families, and health care providers have implications for changes to the way that health care services are provided, in order to better reach men and to involve family members both in a role to support those diagnosed with a condition, and also as a way to increase prevention efforts for those family members who do not already have a diagnosis.

Due to the defined gender roles that were expressed in the interviews in which women largely play the role of caretakers and men are for the most part considered to be wage earners, family-centered strategies and strategies aimed at couples are especially relevant as they present an opportunity to reach the larger family unit.

The primary strength of this study is that it was a study built in to an intervention in a healthy lifestyle program for patients with type 2 diabetes and/or hypertension receiving their care at primary care health centers in Chiapas, Mexico. By building on an intervention study that was underway, the research team was able to pose relevant questions related to the observation of limited participation by men and the missed opportunity to involve family members. This study has several limitations. This study was small in scale; conducting interviews with a larger number of men, family members, and providers would have permitted comparisons between different sub-groups such as those with different levels of disease severity. Interviews were conducted with men, families, and health care providers in one geographic location so the proposed strategies that are presented in this study are primarily relevant to primary care health centers in urban areas of Chiapas, Mexico. However, the family and gender dynamics presented in this study may be similar to other settings in Mexico and throughout the region. The study would have benefited from having included patients receiving care from other health care service providers, in particular the Mexican Social Security Institute, the largest healthcare provider in the country.

### Recommendations for future research and interventions

We agree with others who have called for future research on family-oriented studies, including assessment of outcomes for both the patient and family members, comparison of couple interventions to evidence-based patient interventions, and evaluation of mechanisms of change [25]. Important topics for future research include formative research and intervention studies focused on strategies that involve family members in disease management for chronic conditions. The potential to involve family members both in a support role and as a means to prevent disease in other family members is of increased interest in the United States. The new emphasis in primary care transformation in the United States and specifically the Patient-Centered Medical Home promotes the use of family-centered care strategies, however, in practice, these strategies have not been applied in a systematic way for patients with chronic conditions [26].

Much of the research and practice in involving family members in disease management is primarily focused on the role of being a provider of support to an index member with a chronic condition [10]. However, as we found in this study, families often have more than one family member with a chronic condition and those with chronic conditions often provide support to other family members (e.g. grandchildren, partners, or elder members of the family), especially in multi-generational families. Our study identified what has been reported elsewhere: family interactions may be both a facilitator for and a barrier to disease management, and at times there is no apparent family support [9, 14].

In intervention studies involving families, there is a dearth of relevant family-level measures. This is an area that will require additional research investment, particularly for families with more complex compositions. As was found in this study, genograms and eco-maps can be complementary visual techniques used during interviews with families and may be particularly useful for identifying specific kinds of support and available resources in and outside of the household. We propose their continued use in research studies to complement data gathered during interviews, and also consider that they may be useful as tools in interventions with families.

The potential to prevent disease in other family members has been explored on a limited basis and presents an opportunity to reach a large population. We propose pilot testing interventions at the family-level aiming to increase support for patients with a chronic condition and to promote healthy behavior in other family members. This is particularly relevant when considering pre-diabetes, the increasing obesity epidemic in children, and the fact that children and adolescents have limited control in making decisions about the type of food that they prepare or eat.

A concern is that family-focused strategies may have limited success in engaging the whole family unit, if adult male family members do not take part. As such, family-focused strategies may turn out to mostly involve women and children, in which case the challenge of involving men would persist.

We propose that for future studies, barriers in participation be analyzed separately for men and women, recognizing that their participation in health care and public health strategies often is very different and as such learning the different barriers may lead to different strategies to reach men and women. In-depth ethnographic methods offer a way to understand gender differences, and the family context, in relation to disease management as well as the broader, social context in which individuals and families live [27, 28].

## Conclusions

Men’s participation in cardiovascular health promotion activities offered by primary care health centers was found to be limited by both internal and external factors. Families in which at least one family member had type 2 diabetes and/or hypertension demonstrated diversity in their composition and relationships to the chronic condition. Families ranged from those in which there was apparent support, to others that discussed the opportunity to prevent disease in those currently unaffected, and to other family situations in which support was limited or absent. Men, families, and health care providers recommended numerous and overlapping strategies for involving men and family members.

This qualitative study presents findings relevant for primary care health centers in the capital city of Chiapas, Mexico. Research related to limited uptake of an intervention by certain sub-groups from the population and the topic of involving families in chronic disease management and prevention present important lines of research for future studies, regionally and worldwide.

## Abbreviations

INCAP: Institute of Nutrition of Central America and Panamá
NHLBI: National Heart, Lung, and Blood Institute
